# Supplementary material for: Infection with chikungunya virus confers heterotypic cross-neutralizing antibodies and memory B-cells against other arthritogenic alphaviruses predominantly through the B domain of the E2 glycoprotein
Source: PLoS Negl Trop Dis. 2023 Mar 13;17(3):e0011154. doi: 10.1371/journal.pntd.0011154 (PMC10036167; doi:10.1371/journal.pntd.0011154)
Supplement: S1 Table — Plaque reduction neutralization titer assays were performed to calculate the 50% neutralization titer against a panel of SFV complex alphaviruses and the encephalitic alphavirus VEEV from the endemic cohort (n = 5) and non-endemic cohort (n = 7) at multiple timepoints. The limit of detection was a 1:40 serum dilution and values not determined are denoted with ND due to insufficient serum volume. (DOCX) [file pntd.0011154.s004.docx]

**Supplemental Table 1.** **Compiled PRNT_50_ values for each subject against the six alphaviruses serologically profiled in this study.**

|  | **Subject** | **Years post-infection** | **CHIKV PRNT_50_** | **Una PRNT_50_** | **MAYV PRNT_50_** | **VEEV PRNT_50_** | **ONNV PRNT_50_** | **RRV PRNT_50_** |
| --- | --- | --- | --- | --- | --- | --- | --- | --- |
| **Endemic** | 1 V1 | 2.8 | 8865 | 404 | 383 | 650 | 3648 | 458 |
|  | 1 V2 | 3.3 | 12673 | 488 | 601 | 376 | 5486 | 195 |
|  | 1 V3 | 3.8 | 13612 | 403 | 379 | 56 | 2925 | 126 |
|  | 1 V4 | 6 | 23301 | 1181 | 7661 | <1:40 | 10649 | 204 |
|  | 3 V1 | 4.3 | 8464 | 2400 | 2737 | 43 | 21704 | 479 |
|  | 3 V2 | 5.1 | 28414 | ND | 1080 | <1:40 | 7863 | ND |
|  | 8 | 2.8 | 11834 | 1124 | 519 | <1:40 | 2679 | 182 |
|  | 8 V2 | 3.5 | 25193 | 3300 | 4498 | 150 | 7582 | 261 |
|  | 8 V3 | 5.3 | 12830 | 4119 | 4972 | 122 | 10836 | 322 |
|  | 13 | 3.4 | 59931 | 2400 | 2737 | 43 | 21704 | 479 |
|  | 13 V2 | 4 | 144824 | 8750 | 31764 | 772 | 39498 | 611 |
|  | 14 | 3.4 | 14347 | 1302 | 775 | <1:40 | 6157 | 143 |
| **Non-endemic** | 16 | 1.1 | 17552 | 203 | 1977 | <1:40 | 2727 | 147 |
|  | 16 V2 | 6.9 | 49758 | 521 | 8056 | 171 | 20356 | 210 |
|  | 17 | 24.3 | 82 | <1:40 | 65 | <1:40 | 602 | 1122 |
|  | 18 | 9.3 | 1202 | <1:40 | <1:40 | <1:40 | 409.8 | <1:40 |
|  | 18 V2 | 9.8 | 12523 | <1:40 | 387 | 88 | 2963 | <1:40 |
|  | 18 V3 | 12.4 | 9293 | 54 | 361 | 61 | 3067 | <1:40 |
|  | 19 | 8.75 | 20034 | 96 | 422 | <1:40 | 22546 | 43 |
|  | 19 V2 | 10.4 | 14426 | 98 | 540 | <1:40 | 5683 | <1:40 |
|  | 19 V3 | 11.4 | 5146 | 93 | 1055 | 86 | 7850 | <1:40 |
|  | 20 | 7.9 | 12565 | 178 | 1223 | <1:40 | 59589 | 47 |
|  | 21 | 3.5 | 5996 | 105 | 380 | <1:40 | 907 | <1:40 |
|  | 21 V2 | 7.4 | 15244 | 345 | 1974 | <1:40 | 6332 | 130 |
|  | 22 | 4 | 17924 | 588 | 1025 | <1:40 | 3125 | 171 |
|  | 22 V2 | 8.2 | 36278 | 3698 | 2594 | 173 | 13700 | 119 |
