# Supplementary material for: Infection with chikungunya virus confers heterotypic cross-neutralizing antibodies and memory B-cells against other arthritogenic alphaviruses predominantly through the B domain of the E2 glycoprotein
Source: PLoS Negl Trop Dis. 2023 Mar 13;17(3):e0011154. doi: 10.1371/journal.pntd.0011154 (PMC10036167; doi:10.1371/journal.pntd.0011154)
Supplement: S2 Table — PRNT assays were performed on serum samples incubated with beads alone or beads coupled with E2 B domain protein. PRNT50 values were calculated for each sample using Prism software. Fold change was calculated in Excel and is relative to the appropriate control (Δ1: Fold change in PRNT50 titer following E2B bead treatment relative to non-bead treated serum; Δ2: Fold change in PRNT50 titer following control bead treatment relative to non-bead treated serum). (DOCX) [file pntd.0011154.s005.docx]

**Supplemental Table 2. PRNT_50_ values and fold change of MAYV E2 B domain depleted serum samples relative to controls.**

|  | **MAYV PRNT_50_** | | | | | **CHIKV PRNT_50_** | | | | |
| --- | --- | --- | --- | --- | --- | --- | --- | --- | --- | --- |
|  | **No Beads** | **E2B Beads** | **Control Beads** | **∆1** | **∆2** | **No Beads** | **E2B Beads** | **Control Beads** | **∆1** | **∆2** |
| **Subject 1 v2** | 1424 | 276.8 | 1862 | 0.19 | 1.31 | 28382 | 17945 | 12679 | 0.63 | 0.45 |
| **Subject 3 v2** | 1253 | 347.1 | 1102 | 0.28 | 0.88 | 19932 | 26258 | 25483 | 1.32 | 1.28 |
| **Subject 8** | 2056 | 219.9 | 1621 | 0.11 | 0.79 | 12928 | 10368 | 23924 | 0.80 | 1.85 |
| **Subject 13** | 8319 | 2304 | 10768 | 0.28 | 1.29 | 110426 | 121750 | 127457 | 1.10 | 1.15 |
| **Subject 14** | 1636 | 288.3 | 1470 | 0.18 | 0.90 | 19667 | 11520 | 16025 | 0.59 | 0.81 |
| **Subject 20** | 958.8 | 270.2 | 810.6 | 0.28 | 0.85 | 13466 | 10759 | 11192 | 0.80 | 0.83 |
| **Subject 22** | 2089 | 294.8 | 1134 | 0.14 | 0.54 | 34750 | 36086 | 29079 | 1.04 | 0.84 |
| **Subject 17** | 244.6 | 137.7 | 256.9 | 0.56 | 1.05 | 112.5 | 203.4 | 205.7 | 1.81 | 1.83 |
| **Subject 18** | 110.8 | <100 | 85.8 | N/A | 0.77 | 2961 | 2258 | 3020 | 0.76 | 1.02 |
