# Supplementary material for: Infection with chikungunya virus confers heterotypic cross-neutralizing antibodies and memory B-cells against other arthritogenic alphaviruses predominantly through the B domain of the E2 glycoprotein
Source: PLoS Negl Trop Dis. 2023 Mar 13;17(3):e0011154. doi: 10.1371/journal.pntd.0011154 (PMC10036167; doi:10.1371/journal.pntd.0011154)
Supplement: Attachment — Submitted filename: Review.docx [file pntd.0011154.s007.docx]

**Summary:** Powers et al. used convalescent blood samples from suspected or confirmed CHIKV cases from subjects in endemic and non-endemic areas and found that their antibodies can neutralize several alphaviruses in the Semliki Forest complex. By depleting E2 B domain MAYV specific antibodies from CHIKV subjects, cross-neutralization of MAYV was significantly reduced with no effect on CHIKV neutralization. They also showed that CHIKV-specific memory B-cells exist up to 24 years post infection.

While the authors show that their subjects have neutralizing antibodies against many alphaviruses, there is no evidence to rule out infections by other alphaviruses; so, it is not possible to know if the antibodies are truly cross-neutralizing or generated from natural infection with another alphavirus. This is particularly relevant toward the MAYV E2 domB depletion experiment. The authors suggest that E2 domB could be used for the development of broadly neutralizing alphavirus antibodies, yet the depletion of MAYV E2 domB lead to specific reduction in neutralization only against MAYV and not to CHIKV. These results suggest the opposite—that domB antibodies are virus-specific and will not be broadly neutralizing. A more relevant test to make the claim that CHIKV induced cross-neutralizing antibodies mediated by the E2 domB region would be to deplete CHIKV E2 domB and show an impact on MAYV neutralization. You would have to be sure that the person was only infected with CHIKV, and not MAYV, though, which is challenging with field samples.

The authors also make claims of the longitudinal durability of immunity. The samples are insufficiently powered (n=2) to make such conclusions. Additionally, the authors claim that longitudinal samples demonstrate a reduction in cross-neutralizing antibodies over time but subject 1 v2 has increased titers against CHIKV, UNAV, MAYV, and ONNV and subject 3 v2 also had increased nAb titers for CHIKV, MAYV, and ONNV. Finally, the claim that the samples did not neutralize VEEV is also not supported by the data.

Overall, while the premise of the paper is strong, this manuscript suffers from several limitations. The infection history of the patients cannot be known, casting the CHIKV-specific nature of conclusions in doubt. Further conclusions relating to domain B’s impact, the longitudinal claims, and other interpretations are not supported by the data. The cohorts are interesting and the premise strong. If the authors completely revamp the way the data are interpreted and the story they intend to tell, then there are likely informative data here.

**Other Points:**

1. I am confused by the use of ELISA in the MBC assay, and not PRNT, as was done for the other studies. Please test these samples by PRNT at least against CHIKV and MAYV—and maybe RRV and VEEV.
2. Please add information about the number of independent experiments performed, and the number of technical replicates within each experiment.
3. There are no statistical analysis comparing change in %neutralization between samples
4. It is interesting that depletion of MAYV E2 domB specific antibodies harbored no effect on %neutralization of CHIKV. Given the MAYV titers from patient characterizations in Table 1, is it possible that the they are simply neutralization MAYV specific antibodies?
5. Line 34: Please remove the word “distantly”. I believe it’s misleading since the viruses are relatively closely related, especially compared to CHIKV and VEEV.
6. Line 47: How do you know they’re truly protective responses? Please temper the statements; they are certainly cross-reactive, but PRNTs don’t always predict protection.
7. Line 62: “Puerto Rico recently experienced a CHIKV epidemic starting in May 2014 and official surveillance reported 28,327 suspected cases and 31 deaths by the epidemic’s end” Why only reference PR? I know it’s because you collected samples from PR, but that only becomes clear later in the paper. Please reference the larger CHIKV outbreak in the Americas and then you can highlight the PR part either here or when you reference that your samples are from PR.
8. Line 67: “The most distant member of the SFV complex is Ross River virus (RRV)” Is this true? What is this statement based on? According to Forrester et al. 2012, RRV groups more closely than several other SFV complex members. Please revise.
9. Line 115: Should be “Alphavirus” instead of “Alphaviruses”
10. Line 122: How can it be confirmed that the subjects in the study were only infected with CHIKV or did not have multiple infections with different alphaviruses?
11. Lines 198-199: Conclusions not supported by the data; the neutralization did not decline over time
12. Lines 201-206: RRV subject should be excluded. Including the RRV subject of n=1 is not sufficient for offering “novel information about likely RRV cross-reactive(-ity)….”
13. Lines 133-134 posits that cross-reactive nAb levels demonstrate time-dependent reduction – this is not true: subject 1 experience increased nAb titers for CHIKV, UNAV, MAYV, and ONNV while subject 3 experiences increased nAbs titers for CHIKV, MAYV, and ONNV.
14. Lines 144-145 suggest that longitudinal samples from subjects 1 and 3 responses indicate a reduction in cross-neutralizing antibodies. The data do not support this (additionally, we cannot rule out re-infections so postulating the waning of abs seems speculative)
15. Line 154: The claim regarding the binding pocket in Figure 3C is speculative and unclear as the photo lacks annotations
16. Line 161: How can we be sure that the antibodies we depleted are MAYV cross-reactive antibodies and not MAYV-specific antibodies? If the depleted antibodies were CHIKV antibodies that also bound MAYV, shouldn’t the CHIKV PRNT change?
17. Line 178: The range of cross-reactive is very large (1.7% to 98%), and they are referred to as specific MBCs. Does this mean that the people were infected with MAYV?
18. Line 230: I disagree that these results can be used to establish an infection history. It is unclear what the infection history was for any of these patients. This study was also not set up correctly to draw these conclusions.
19. Line 262: Which viruses were grown in which cells? This matters, especially since the viruses are being compared to each other. What MOI was used to propagate the viruses? Viruses grown in mosquito cells will have different glycosylation patterns and likely neutralization results than those grown in mammalian cells. How might this technical decision impact the PRNT results?
20. Line 267: Please define PSG.
21. Line 291: “MAYV BeAr505411 was reversed transcribed using SuperScript IV Reverse Transcriptase and cloned into pcDNA3.1.” Please clarify this statement. I assume you mean the RNA was reverse transcribed, then you did PCR for the domain B region, and then you cloned it.
22. Line 378: The PMIDs should be proper citations
