# Supplementary material for: Infection with chikungunya virus confers heterotypic cross-neutralizing antibodies and memory B-cells against other arthritogenic alphaviruses predominantly through the B domain of the E2 glycoprotein
Source: PLoS Negl Trop Dis. 2023 Mar 13;17(3):e0011154. doi: 10.1371/journal.pntd.0011154 (PMC10036167; doi:10.1371/journal.pntd.0011154)
Supplement: Attachment — Submitted filename: Rebuttal PLosNTD Manuscript.docx [file pntd.0011154.s008.docx]

**RE: Infection with chikungunya virus confers heterotypic cross-neutralizing antibodies and memory B-cells against other arthritogenic alphaviruses predominantly through the B domain of the E2 glycoprotein**

First, we would like to thank the reviwers for their careful evaluation of our manuscript and apologize for the length of time that it took to get our manuscript back for re-review. As you will notice our updated version contains a considerable amount of new data that, by the nature of the experiments, took a long time to complete. For example two major additions include: 1) the procurement of additional plasma samples collected from our cohort that provides additional longitudinal information (time points listed in **Table 1**); and 2) we engineered a CHIKV-E2B construct and produced protein for Ab depletions (**Supplemental Figure 1C and D**) and neutralization assays were performed similarly to experiments previously described with MAYV E2B (**Figure 4-neut data; Figure 5C and D-fold change data; Supplemental Table 3 summarizes data**). We feel strongly that the new data and analyses provide support of our original conclusions and provide another level of validation and Figure 5 now includes additional analysis of CHIKV E2B vs. MAYV-E2B depletion results; and Figures 5E and F provides an additional level of analysis. To this end, we have added the new samples data to **Figure 1B and C**, and **Figure 1D** provides a summary of all of the samples in regard to alphaviral neutralization breadth and contains appropriate statistical analysis. This data adds depth to the analysis and replaces previous **Figure 7A**, which is no longer be included in the manuscript. Similarly F**igure 7B** was added to **Figure 6** (current panel D). Additional longitudinal sample neutralization breadth information has been added to the antigenic cartography results shown in **Figure 2**, and we now provide two panels depicting either only the initial sampling time points for each subject (**Figure 2A**) or the impact of longitudinal sampling that is depicted in **Figure 2B**. Data previously shown as a table within **Figure 6** (previous panel D) was converted to **Table 2** for ease of readership and to follow PLoS guidelines. These changes and text edits suggested by the reviewers are highlighted in the text for ease of review. A rebuttal to the specific questions posed by the reviewers is provided below.

**----------------------------------------------------------------------------------------------------------**

**Reviewer #1**

1. Lines 141 and 143: There is no Fig 2A. Do the authors’ mean to say Fig. 1A or Figs. 1A and 2?

Thank you for this feedback, we have updated the text accordingly to match the figures. The line now reads “We found that CHIKV and ONNV are the most antigenically similar, consistent with the phylogenetic analysis (Fig 1A)…”

2. Line 434, legend to Fig 3: There is no 'D' panel shown in the Fig. 3.

Thank you for this feedback, we have corrected the figure to reflect panels A-D.

3. The antibody depletion data are particularly interesting, but I feel that they could have been made more robust with analyses of the E2B-bead captured antibodies. It should be possible to elute such E2B-specific antibodies from the beads and analyse their anti-MAYV neutralization potency in more detail.

Thank you for this, we agree that it would be interesting to comprehensively interrogate the antibodies that bound to MAYV E2B and plan to do this in the future. We now provide data demonstrating the ability of CHIKV E2B protein depletion of cross reactive antibodies that neutralize MAYV.

4. While the authors show that their subjects have neutralizing antibodies against many alphaviruses, there is no evidence to rule out infections by other alphaviruses; so, it is not possible to know if the antibodies are truly cross-neutralizing or generated from natural infection with another alphavirus. This is particularly relevant toward the MAYV E2 domB depletion experiment. The authors suggest that E2 domB could be used for the development of broadly neutralizing alphavirus antibodies, yet the depletion of MAYV E2 domB lead to specific reduction in neutralization only against MAYV and not to CHIKV. These results suggest the opposite—that domB antibodies are virus-specific and will not be broadly neutralizing. A more relevant test to make the claim that CHIKV induced cross-neutralizing antibodies mediated by the E2 domB region would be to deplete CHIKV E2 domB and show an impact on MAYV neutralization.

The authors thank the reviewer for this feedback, and we now present our new analysis of antiserum that were depleted using CHIKV E2B. Post depletion neutralization assays were performed against CHIKV and MAYV (Figure 4 and fold differences quantified in figure 5C and D). We found that CHIKV-E2B depletion results in a reduction in heterotypic neutralization and to a much lesser extent homotypic neutralization. Indicating that antibodies targeted to the E2B domain play an important role in cross-neutralization.

5. You would have to be sure that the person was only infected with CHIKV, and not MAYV, though, which is challenging with field samples.

The authors appreciate this comment, it is difficult to accurately determine infection history after the fact. All 5 endemic subjects were confirmed by PCR during acute CHIKV infection. None of the 7 endemic subjects had a confirmed CHIKV infection, they all had documented undiagnosed febrile illness, travel history consistent with travel to endemic regions where CHIKV circulates and a CHIKV-specific 50% PRNT titer of >1:20. Only one subject, Subject 16, encountered their alphavirus infection in the Central American country of El Salvador, a region where MAYV circulates. However, based on serology data, the highest neutralizing antibody titers were against CHIKV and >4-fold higher than MAYV, we called this subject primary CHIKV immune. However, we surmise that Patient 17 was infected in Papua New Guinea with RRV and not CHIKV based upon their serological profile.

6. The authors also make claims of the longitudinal durability of immunity. The samples are insufficiently powered (n=2) to make such conclusions.

We agree with this comment and have collected additional longitudinal samples for a total of nine subjects with at least two serum samples and a subgroup with three time points (Table 1; Figure 1B and C).

7. Additionally, the authors claim that longitudinal samples demonstrate a reduction in cross-neutralizing antibodies over time but subject 1 v2 has increased titers against CHIKV, UNAV, MAYV, and ONNV and subject 3 v2 also had increased nAb titers for CHIKV, MAYV, and ONNV. Finally, the claim that the samples did not neutralize VEEV is also not supported by the data.

As indicated above, we have added additional longitudinal samples. The original observations were based on cross-sectional analysis looking at the cohort as a whole, when we look longitudinally within subjects we indeed do not see a reduction in cross-neutralizing antibody titers, instead all of the antibody populations appear relatively stable over the time period observed. We have reworded the discussion to better align with the data. Line 241 reads “Broad neutralization was observed in both endemic and non-endemic subjects with antibody breadth against antigenically distinct viruses remaining stable over time (Fig. 1).”

8. I am confused by the use of ELISA in the MBC assay, and not PRNT, as was done for the other studies. Please test these samples by PRNT at least against CHIKV and MAYV—and maybe RRV and VEEV.

We appreciate this comment; however, conducting the MBC *ex vivo* stimulation results in a limited sample volume. There was simply not enough volume to perform more assays. The authors looked for MBC-derived antibodies that bound to CHIKV, MAYV, and MAYV E2B and used this information to calculate a frequency of antigen-specific and cross-reactive circulating MBCs in each subject.

9. Please add information about the number of independent experiments performed, and the number of technical replicates within each experiment.

Additional details were added to methods and figure legends to indicate the number of replicates in each experiment.

10. There are no statistical analysis comparing change in %neutralization between samples

This analysis has been added to Figure 1 and presented in panel D.

11. It is interesting that depletion of MAYV E2 domB specific antibodies harbored no effect on %neutralization of CHIKV. Given the MAYV titers from patient characterizations in Table 1, is it possible that the they are simply neutralization MAYV specific antibodies?

We agree that with MAYV-E2B depletions alone the data could indicate this; however, with the addition of CHIKV-E2B depletion experiments that show a similar trend to MAYV-E2B depletion but limited effect on CHIKV neutralization, we believe this strongly suggests that E2B binding antibodies are important for heterotypic-neutralization and other epitopes in E2 or E1 are critical to establish a wide breadth of targets directed against the homotypic virus neutralization.

12. Line 34: Please remove the word “distantly”. I believe it’s misleading since the viruses are relatively closely related, especially compared to CHIKV and VEEV.

The line now reads “We functionally assessed the ability of memory B-cell derived antibodies to bind to chikungunya virus, and related Mayaro virus…”

13. Line 47: How do you know they’re truly protective responses? Please temper the statements; they are certainly cross-reactive, but PRNTs don’t always predict protection.

We agree that neutralizing antibodies are not always predictive of protection, the line now reads “Our results indicate that infection with chikungunya virus results in a robust and durable cross-reactive humoral immune response. One that could potentially provide immunity against repeat infection with chikungunya as well as related alphaviruses for years to decades after initial infection.”

14. Line 62: “Puerto Rico recently experienced a CHIKV epidemic starting in May 2014 and official surveillance reported 28,327 suspected cases and 31 deaths by the epidemic’s end” Why only reference PR? I know it’s because you collected samples from PR, but that only becomes clear later in the paper. Please reference the larger CHIKV outbreak in the Americas and then you can highlight the PR part either here or when you reference that your samples are from PR.

Thank you for pointing this out. The introduction now includes a more detailed background on the emergence of CHIKV in the Americas lines 70 – 78 reads “Before 2013 CHIKV had not yet been locally acquired or transmitted within the Americas [7]. Historically, circulating predominantly in regions of Africa and Asia, but emerged on a globally scale in the mid-2000s, resulting in outbreaks in Africa, Asia, as well as the Caribbean and North, Central, and South Americas, resulting in almost 2 million reported infections [8]. At the time of writing (October 27th 2022), there have been 338,592 cases with 70 deaths in 2022, with the majority of the cases occurring in Brazil (ECDC). In the current investigation, we characterize samples from an endemic human cohort in Puerto Rico. The island of Puerto Rico experienced a CHIKV epidemic starting in May 2014 with official surveillance reporting 28,327 suspected cases and 31 deaths by the epidemic’s end [9].”

15. Line 67: “The most distant member of the SFV complex is Ross River virus (RRV)” Is this true? What is this statement based on? According to Forrester et al. 2012, RRV groups more closely than several other SFV complex members. Please revise.

We have revised our statement, the line now reads “The most distant member of the SFV complex that we included in our alphavirus panel is Ross River virus (RRV), which is endemic to Australia and several neighboring Pacific Islands [5].”

16. Line 115: Should be “Alphavirus” instead of “Alphaviruses”

This has been corrected, the line now reads “Alphavirus specific neutralization and antigenic relationship by subject.”

17. Line 122: How can it be confirmed that the subjects in the study were only infected with CHIKV or did not have multiple infections with different alphaviruses?

As raised above, it is difficult to accurately determine infection history after the fact. All 5 endemic subjects were confirmed by PCR during acute CHIKV infection with no additional febrile illness reported. None of the 7 endemic subjects had a confirmed CHIKV infection, they all had documented undiagnosed febrile illness, travel history consistent with regions where CHIKV circulates and a CHIKV-specific 50% PRNT titer of >1:20. Only one subject, Subject 16, encountered their alphavirus infection in the Central American country of El Salvador, a region where MAYV circulates. However, based on serology data, highest neutralizing antibody titers were against CHIKV and >4-fold higher than MAYV, we called this subject primary CHIKV immune.

18. Lines 198-199: Conclusions not supported by the data; the neutralization did not decline over time

To address this Lines 239-243 now reads “Our data highlights that primary infection with CHIKV not only elicits durability of long-term homotypic neutralizing antibodies, up to 24 years after infection, but also induces neutralizing antibody breadth that extends across multiple SFV complex alphaviruses. Broad neutralization was observed in both endemic and non-endemic subjects with antibody breadth against antigenically distinct viruses remaining stable over time (Fig. 1).”

19. Lines 201-206: RRV subject should be excluded. Including the RRV subject of n=1 is not sufficient for offering “novel information about likely RRV cross-reactive(-ity)….”

We chose to retain this subject because they were initially identified by our anti-CHIKV serological screen. While we agree that an n=1 is not enough to support a novel claim, this is the nature of doing human cohort studies in wide geographic ranges where it is understood that many different alphaviruses circulate. Our finding supports the need for more in-depth screening and highlights the difficulty of accurately determining infection history by serology alone. The wording has been changed to better illustrate our point. It now reads “During this investigation it was determined that one of our alphavirus immune subjects (Subject 17) had a serum neutralization profile more consistent with prior RRV instead of CHIKV infection. This conclusion is consistent with travel history (infected in Papua New Guinea) and by antigenic cartography (Fig. 2A) where the subject clustered most closely to RRV antigenically. This subject was initially identified through a CHIKV specific neutralization assay screen, and we chose to retain the subject in our analyses because their infection history adds information about the breadth of Ab responses within the SFV-complex and illustrates the importance of specific serological tests to determine infection history.”

20. Lines 133-134 posits that cross-reactive nAb levels demonstrate time-dependent reduction – this is not true: subject 1 experience increased nAb titers for CHIKV, UNAV, MAYV, and ONNV while subject 3 experiences increased nAbs titers for CHIKV, MAYV, and ONNV.

We have reworded the results section, line 163-167 now reads ”Overall for 9 of the 12 subjects that we serologically profiled at more than one time point post-infection, we found longitudinal changes in both homotypic and heterotypic neutralization over time to be variable compared to the original blood draw but overall antibodies remained stable overtime (Fig 1B,C).”

21. Lines 144-145 suggest that longitudinal samples from subjects 1 and 3 responses indicate a reduction in cross-neutralizing antibodies. The data do not support this (additionally, we cannot rule out re-infections so postulating the waning of abs seems speculative).

We have increased the number of longitudinal samples analyzed in this study (n=9) to address this comment about the durability of cross-neutralizing antibodies. We have re-written the results to read “All sera tested cluster around CHIKV and the closely related ONNV, except for Subject 17, which clusters most closely to RRV (Fig 2A). Longitudinal samples (Fig. 2B) continue to cluster most closely to CHIKV suggesting maintenance of CHIKV-specific and cross reactive antibodies over time (Fig 2B).” To address the waning antibody question, we looked at 2 distinct cohorts of subjects, a non-endemic and an endemic. We might expect to see re-exposure/re-infections in our endemic environment, but not in our non-endemic cohort. During the course of this study, we did not observe differences in antibody dynamics over time between our endemic and non-endemic subjects.

22. Line 154: The claim regarding the binding pocket in Figure 3C is speculative and unclear as the photo lacks annotations

We have rewritten this statement to include “When viewed in a structural model, the organization of the E1-E2 monomer and arrangement in the spike trimer demonstrates the accessibility of antibody binding to the E2B domain (Fig 3C and D).” The intent was to illustrate that the E2B domain is highly accessible.

23. Line 161: How can we be sure that the antibodies we depleted are MAYV cross-reactive antibodies and not MAYV-specific antibodies? If the depleted antibodies were CHIKV antibodies that also bound MAYV, shouldn’t the CHIKV PRNT change?

With MAYV-E2B depletions alone the data could indicate that the antibodies were in fact MAYV-specific; however, with the addition of CHIKV-E2B depletion experiments (Fig. 4 and 5C and D) also showed a similar trend to MAYV-E2B depletion with regards to MAYV and CHIKV neutralziation. We observe a decrease in heterotypic neutralization titers (MAYV) and relatively little change in homotypic (CHIKV) neutralization titers. We believe this strongly suggests that E2B binding antibodies are important for heterotypic-neutralization but the nature of infection to elicit Abs targeting additional viral epitopes determines the specificity of homotypic neutralization.

24. Line 178: The range of cross-reactive is very large (1.7% to 98%), and they are referred to as specific MBCs. Does this mean that the people were infected with MAYV?

We described the range of E2B MBC-derived antibodies attributable to MAYV-specific antibodies, and we reworded this for clarification. The line now reads “The variability of cross-reactive MBCs attributable to E2B varied by subject (Fig 6D), ranging from 1.7% to 98% of MAYV-binding MBCs.

25. Line 230: I disagree that these results can be used to establish an infection history. It is unclear what the infection history was for any of these patients. This study was also not set up correctly to draw these conclusions.

In line 230 we state “…as well as point out the importance of specific serologic assays to determine alphavirus infection histories”. We were simply stating that because of the broad cross-reactivity observed within the SFV complex it is essential to preform specific serology tests or develop more accurate tests to be able to distinguish between these closely related infections.

26. Line 262: Which viruses were grown in which cells? This matters, especially since the viruses are being compared to each other. What MOI was used to propagate the viruses? Viruses grown in mosquito cells will have different glycosylation patterns and likely neutralization results than those grown in mammalian cells. How might this technical decision impact the PRNT results?

All of the viruses used in this study were propagated in C6/36 mosquito cells in the same manner as described in the methods, lines 313 – 324 using an MOI = 0.01. The titer and neutralization assays were completed in Vero cells. Since we did not compare virus grown in mammalian cells with the insect cell derived virus, we can not speculate as to the effect it might have on the PRNT results. We would consider looking at this further in a new study.

27. Line 267: Please define PSG.

We have defined this in the text: Penicillin Streptomycin Glutamine (PSG).

28. Line 291: “MAYV BeAr505411 was reversed transcribed using SuperScript IV Reverse Transcriptase and cloned into pcDNA3.1.” Please clarify this statement. I assume you mean the RNA was reverse transcribed, then you did PCR for the domain B region, and then you cloned it.

The methods were re-written as follows “cDNA was synthesized using SuperScript IV Reverse Transcriptase (Invitrogen) and the MAYV E2 B domain was amplified with forward primer TGAATTCCATATG-HiBit GTGAGCGGCTGGCGGCTGTTCAAGAAGATTAGC-CCGGACATTCCGGATAGAAC and reverse primer AAGCTTTTAGTGATGGTGATGGTGATGGCTCGTGACGTAAGCCTGACATTTG and cloned into pcDNA3.1.”

29. Line 378: The PMIDs should be proper citations

Thank you for pointing this out, the references have been properly formatted.

**Reviewer 3.**

1. Line 161 and Figure 4. Please show the non-linear regression / neutralizing activity curve used to determine the IC50 titer. Showing the IC50 titers or other descriptive readout, such as AUC, would be informative, especially if you perform statistical analyses on those derived data points between groups. I understand you show statistical analyses of the fold changes in figure 5 but distilling the data in figure 4 to a statistical comparison between groups would strengthen the conclusions around figure 4.

Thank you for this feedback, we have provided the PRNT_50_ values following depletion in Supplemental Tables 2 and 3. The neutralization curves used to calculate the PRNT50 values are shown in Figure 4. The lines used for the non-linear regression were not included because it adds another four lines to the graphs and makes them difficult to interpret. Howerver, we provide the Regression curves below. These figures could be added to the paper if the reviewer feels that this is required for full interpretation. Indeed, Figure 5 includes statistical analysis of the fold change in neutralization from figure 4. We agree that another statistical comparison of PRNT_50_ between treatment groups will strengthen our conclusions, so we have included additional panels in Figures 5E and 5F to compare changes in neutralization from the depletion experiment with grouping by subject. Consistent with the fold change data and statistical analysis, we found that the comparison of MAYV E2B depletion to control beads for MAYV neutralization resulted in a significant decrease in PRNT_50_. Effects on CHIKV titer or use of control beads for depletion were found to be statistically insignificant. The authors confer that this additional analysis showing the significant impact of MAYV E2B depletion on MAYV cross-neutralization paired with the fold change data is sufficient to support a role for E2B in cross-neutralization.

2. Figure 6. What are the black dots?

The black dots correspond to naïve subjects, described in the figure legend that reads “Antigen-specific MBC frequency (per 10^6^ PBMC) over time in non-endemic cohort (blue n=6), endemic (orange n=5), and naïve subjects (black n=3).”

3. Figure 7A. Is this just a different analysis of the same data from figure 1b? If I am understanding correctly, figure 7 is the data used to justify the conclusion that MBCs are responsible for much of the cross-neutralizing antibody responses. If this is correct, it may be better to report fold-change on the y axis and just show both the total nAb and MBC-specific data on one graph. Otherwise the MBC argument is harder to grasp by the reader.

We have removed Figure 7 from the manuscript. We included the neutralization data (formally Figure 7A) in a larger analysis and comparison of cross-neutralizing antibody titers grouped by subject, found in Figure 1D. The MBC data (formally Figure 7B) has been incorporated into Figure 6.

4. Line 207. For the conclusion that CHIKV-E2 B binding antibodies, elicited by CHIKV infection, contribute to cross-neutralization, depletion only of MAYV-specific E2 B binders seems counterintuitive. This begs the question, what would depletion of MAYV-specific non-E2 B binders look like in terms of MAYV neutralization? My opinion is that you would need both data sets to strengthen the conclusion that E2 B binding antibodies contribute more to cross-neutralization than any other epitope. The simplest explanation for this data is that if you deplete any MAYV-specific binders, you will see reduction in MAYV neutralization. This conclusion is strengthened by the observation that depletion of MAYV-specific E2 B binders does not impact CHIKV neutralization, suggesting that other epitopes contribute significantly to neutralization. Alternatively, if you had depleted CHIKV-specific E2 B binding antibodies and demonstrated reduction in MAYV neutralization, then the conclusion would be better supported since you are not depleting MAYV-specific binders. Happy to be wrong on this of course and welcome any rebuttal!

We appreciate this helpful feedback and thoughts, and as such we performed the suggested experiment to deplete serum of CHIKV E2B binding antibodies (shown in Fig. 4 and 5C and D). We observed similar results to MAYV and CHIKV neutralization titers that we determined following MAYV-E2B depletion. We determined a greater loss of heterotypic (MAYV) neutralization titer when compared to homotypic neutralization titers; we believe these results further support our claim that E2B-specific antibodies are important for cross-neutralization between CHIKV and MAYV while other epitopes are important for homotypic neutralization.

5. It would be helpful to the reader if you could re-iterate your overall conclusions for each figure at the end of each results section. A lot of the paper seems to be descriptive without the conclusions statements to apply a lot of the descriptive observations.

Thank you for this feedback, we have added a conclusion statement to each section in the results as well as additional conclusions statements to the discussion.
