# Supplementary material for: Infection with chikungunya virus confers heterotypic cross-neutralizing antibodies and memory B-cells against other arthritogenic alphaviruses predominantly through the B domain of the E2 glycoprotein
Source: PLoS Negl Trop Dis. 2023 Mar 13;17(3):e0011154. doi: 10.1371/journal.pntd.0011154 (PMC10036167; doi:10.1371/journal.pntd.0011154)
Supplement: Attachment — Submitted filename: Review.docx [file pntd.0011154.s009.docx]

**Summary:** Powers et al. describe a cohort of non-endemic and endemic patients with suspected or confirmed primary CHIKV infections and assessed long-term and cross-neutralizing antibodies and memory B-cells against other alphaviruses, particularly against MAYV. Their data suggest that CHIKV neutralizing antibodies are cross-neutralizing for MAYV, ONNV, UNAV, and RRV but not VEEV – postulating that antibodies binding to E2B domain mediate the cross protection. Allow the study design, samples sources, and hypotheses are all compelling, several concerns remain or have been identified during the review.

**Major points:**

1. Line 225: Based on your data, it appears that homotypic E2B antibodies don’t neutralize CHIKV. This result is inconsistent with prior literature. How did you confirm that the CHIKV E2B protein was functional? I assume the positive control would have been to deplete antibodies and show that neutralization against CHIKV was altered – that wasn’t the case, though. The difference in synthesis of the MAYV-E2B and the CHIKV-E2B may be responsible.
2. Line 267: Based on your data, can you truly say that the patients were only “primary” CHIKV patients? This would imply that they had never been reinfected, but are you sure this is true? Some of the PRNT titers to the other alphaviruses rise over time, suggesting they may have been infected with another virus.
   a. Specifically, patients 1, 3, 13, 16, 21, and 22 all have increasing neutralization titers. Does this not imply that the patients were re-infected with an alphavirus? Patients 1 and 13 have a very stark increase in MAYV titers, indicating that they may have been infected with MAYV at a date between the two blood draws.
   b. It would be beneficial to claim that “neutralizing antibodies are maintained for years after CHIKV infection” and make no claims regarding protection. Furthermore, the authors should highlight the possibilities of re-exposure to an alphavirus in several patients.
3. Line 282: “When serum was depleted of CHIKV or MAYV E2 B domain binding antibodies, cross-neutralization was significantly ablated without significantly reducing neutralization against CHIKV.” But there is a significant difference for CHIKV in Fig 5d, although it doesn’t look like there should be.
4. Related point to #4: Upon further analysis of the neutralization data from either MAYV-E2B and CHIKV-E2B depletions in Figure 5A-D and the supplementary, there is concern on the method of fold change used in the manuscript. Firstly, Δ1 and Δ2 are not defined, however, upon calculation it is observed that Δ1 was calculated as (no beads/E2B) and given and arbitrary (-) negative sign to indicate a decreased in neutralization. Fold change should not be represented as negatives, rather, increase or decreased results should be less than or greater than one. Furthermore, Δ2 is calculated different as (control beads/no beads), also given negative values. These results are directly compared in the text and is misleading given the different analyses. This is particularly of note considering the authors statistical analyses differ when assessing fold-change compared to changes in PRNT50 for CHIKV-E2B depletion on CHIKV.
5. Line 293: “Overall, this indicates that there is greater cross-reactivity observed in MBC-derived antibodies versus serum antibodies.” But they’re also very different assays. Couldn’t the difference between neutralizing and binding antibodies also explain this difference?
6. Line 309: “The durability and breadth of the B-cell mediated immune response to CHIKV indicates that regions with high CHIKV seroprevalence constrict the range of closely related alphaviruses as well as point out the importance of specific serologic assays to determine alphavirus infection histories.” How do you know that these people would be protected from a MAYV infection? This statement is far too strong.

**Other Points:**

1. Line 28: should be “chikungunya virus infections”
2. Line 34: How do you define highly conserved? Presumably the domain B of E2 would be less conserved than nsp4, for example. Given that it’s the major target for antibodies, I would guess domain B is also less conserved than domain C of E2.
3. Line 52: This is nitpicky, but given viruses like Eilat, the statement that alphaviruses are all arboviruses is untrue.
4. Line 58: please provide a citation that SFV infection is predominately associated with myalgia and arthralgia. SFV is kind of an exception to the rule here.
5. Line 115: Change alpahviruses to alphavirus
6. Line 119: Please provide a rationale for making a tree that includes only E1, 6K, and E2 (probably should be written as E2, 6K, and E1). Why not include E3?
7. Lime 132: “Interestingly, this person still had heterotypic immunity even at >20 years post infection.” Or was infected with both viruses? Seems like unnecessary speculation.
8. Line 150: What is the basis for 56% being considered highly conserved? Compared to VEEV, I suppose it’s true, but compared to other viral proteins, I would guess it’s less conserved
9. Line 161-162: How do you define “long-lasting”?
10. Please define the orange and blue dots in Fig. 6 in the figure itself.
11. Line 179: I don’t quite understand how both of these statements can be true: “We found that CHIKV and MAYV-MBC frequencies were highly correlated (R2=0.747) **with a ratio of CHIVK:MAYV of about 10:1 overall**” and **“we observed about a four-fold difference in CHIKV versus MAYV MBC frequencies** (9.7 compared to 2.5)”. I imagine I’m understanding something incorrectly. Can you please clarify how these comparisons are different?
12. Line 210: You refer to homotypic immunity. I believe you are referring to homotypic immunity against CHIKV, but please clarify this.
13. “Our data expand on previously published data by Zhang et. al. that identified that amino acids in the E2 A and B domains are involved in the binding of CHIKV to both mouse and human Mxra8, implicating these domains as major targets for therapeutic strategies” I don’t understand the connection here. The current manuscript has nothing to do with Mxra8 other than being a receptor for CHIKV. I don’t understand the inclusion of this sentence.
14. The rationale for retaining subject 17 is still unclear. Including this subject in all studies assessing if CHIKV sera generates cross-neutralizing antibodies and MBCs skews the data.
15. The arrangement of figures and how they are discussed non-chronologically, particularly figure 4 and 5, make the manuscript hard to follow.
16. Figure 2: The cartography is an interesting analysis approach; however, it is a little confusing to follow. I would recommend the author moves this to supplemental figures.
17. Figure 3: I recommend that authors remove panels C and D, as the protein structures are not novel information, nor are they easy to understand.
18. Figure 4: I recommend the authors swap the supplemental table that highlights the PRNT50s from these data and the graphs, moving the graphs to supplemental data and the tables to the main text.
19. Figure 5: Panels A-D should be reanalyzed. It is not possible to get a negative number for fold change. I suggest swapping the order of Panels E/F with A-D – this will improve the overall flow.
