# Supplementary material for: Infection with chikungunya virus confers heterotypic cross-neutralizing antibodies and memory B-cells against other arthritogenic alphaviruses predominantly through the B domain of the E2 glycoprotein
Source: PLoS Negl Trop Dis. 2023 Mar 13;17(3):e0011154. doi: 10.1371/journal.pntd.0011154 (PMC10036167; doi:10.1371/journal.pntd.0011154)
Supplement: Attachment — Submitted filename: Rebuttal to Reviewers Comments.docx [file pntd.0011154.s010.docx]

**Rebuttal to Review of Manuscript PNTD-D-22-00103R1**

We would like to thank the editor and reviewers for their thorough review of our resubmitted manuscript and for their thoughtful comments on how to improve the paper. We appreciate that the reviewers felt that our additional data that was added following the primary review strengthened the paper. Below we have addressed Reviewer #2’s concerns.

**Major points:**

1. Line 225: Based on your data, it appears that homotypic E2B antibodies don’t neutralize CHIKV. This result is inconsistent with prior literature. How did you confirm that the CHIKV E2B protein was functional? I assume the positive control would have been to deplete antibodies and show that neutralization against CHIKV was altered – that wasn’t the case, though. The difference in synthesis of the MAYV-E2B and the CHIKV-E2B may be responsible.

*We thank the reviewer for helping us realize that we need to clarify this point. First, our results are consistent with published findings that indicate that CHIKV homotypic neutralization is occurring due to antibodies that recognize epitopes in addition to those found in E2B (e.g., the remainder of E2 and E1). Thus, we are suggesting that the homotypic antibody response to CHIKV is broadly targeting whereas the majority of the heterotypic antibody response to MAYV is targeting E2B. Second, the E2B region is a region that is exposed on the surface of the virion and lays in a conformation that is highly accessible to antibody binding, our polypeptide is not meant to be functional for anything other than fishing out antibodies that bind to it. But it turns out that the E2B polypeptides were highly functional in that we were able to deplete antibodies that bound to either CHIKV E2B or MAYV E2B that had the effect of reducing neutralization capacity of that serum against MAYV. The polypeptides were synthesized using the same strain of bacteria, plasmid expression system, and purification scheme and we would not expect significant differences in the synthesis.*

2. Line 267: Based on your data, can you truly say that the patients were only “primary” CHIKV patients? This would imply that they had never been reinfected, but are you sure this is true? Some of the PRNT titers to the other alphaviruses rise over time, suggesting they may have been infected with another virus.
   a. Specifically, patients 1, 3, 13, 16, 21, and 22 all have increasing neutralization titers. Does this not imply that the patients were re-infected with an alphavirus? Patients 1 and 13 have a very stark increase in MAYV titers, indicating that they may have been infected with MAYV at a date between the two blood draws.
   b. It would be beneficial to claim that “neutralizing antibodies are maintained for years after CHIKV infection” and make no claims regarding protection. Furthermore, the authors should highlight the possibilities of re-exposure to an alphavirus in several patients.

*We have removed the word “primary” as the reviewer is correct that we have no proof that the subjects were only infected once or whether they were infected with additional alphaviruses. We have also added a statement “Broad neutralization was observed in both endemic and non-endemic subjects with antibody breadth against antigenically distinct viruses remaining stable over time (Fig. 1); although we also recognize that the subjects could have been infected, even subclinically, with CHIKV or another alphavirus and this might impact Ab responses to CHIKV or other alphaviruses.* *Antibody responses do vary over time probably dependent upon how the adaptive immune response is being challenged at the time of blood collection, and this may not have anything to do with alphavirus infection. We agree that some of the responses go up a little, but others also drop. We would argue that these changes are generally not very significantly different. The point of this analysis was to determine whether the Ab responses were maintained over time and they appear to be, according to our data.*

3. Line 282: “When serum was depleted of CHIKV or MAYV E2 B domain binding antibodies, cross-neutralization was significantly ablated without significantly reducing neutralization against CHIKV.” But there is a significant difference for CHIKV in Fig 5d, although it doesn’t look like there should be.

*We did observe a slightly larger decrease in CHIKV neutralization by antibodies depleted with the CHIKV E2B vs. MAYV E2B, this would be predicted based on the neutralization importance of the E2B region. As described below in Point #4, upon data correction, this difference is still significant suggesting anti-E2B antibodies contribute to the overall neutralization titer for CHIKV.*

4. Related point to #4: Upon further analysis of the neutralization data from either MAYV-E2B and CHIKV-E2B depletions in Figure 5A-D and the supplementary, there is concern on the method of fold change used in the manuscript. Firstly, Δ1 and Δ2 are not defined, however, upon calculation it is observed that Δ1 was calculated as (no beads/E2B) and given and arbitrary (-) negative sign to indicate a decreased in neutralization. Fold change should not be represented as negatives, rather, increase or decreased results should be less than or greater than one. Furthermore, Δ2 is calculated different as (control beads/no beads), also given negative values. These results are directly compared in the text and is misleading given the different analyses. This is particularly of note considering the authors statistical analyses differ when assessing fold-change compared to changes in PRNT50 for CHIKV-E2B depletion on CHIKV.

*We apologize that the spreadsheet that we used had a mistake, the revised data set is now included in the manuscript. The new data has proper fold change values (non-negative numbers).*

5. Line 293: “Overall, this indicates that there is greater cross-reactivity observed in MBC-derived antibodies versus serum antibodies.” But they’re also very different assays. Couldn’t the difference between neutralizing and binding antibodies also explain this difference?

*We agree and have removed this sentence.*

6. Line 309: “The durability and breadth of the B-cell mediated immune response to CHIKV indicates that regions with high CHIKV seroprevalence constrict the range of closely related alphaviruses as well as point out the importance of specific serologic assays to determine alphavirus infection histories.” How do you know that these people would be protected from a MAYV infection? This statement is far too strong.

*We agree and we have softened this statement to reflect that sero-cross reactivity may impact alphavirus in a population. We also address this topic in the discussion at line 274.*

**Other Points:**

1. Line 28: should be “chikungunya virus infections”

*We have modified this sentence*.

2. Line 34: How do you define highly conserved? Presumably the domain B of E2 would be less conserved than nsp4, for example. Given that it’s the major target for antibodies, I would guess domain B is also less conserved than domain C of E2.

*The specific levels of E2b sequence identity is listed in the Results paragraph: Dissecting the role of E2 B domain in homotypic and heterotypic neutralization and in Figure 3A & B.*

3. Line 52: This is nitpicky, but given viruses like Eilat, the statement that alphaviruses are all arboviruses is untrue.

*We agree that not all alphaviruses are arboviruses but fail to identify where this is stated in the text.*

4. Line 58: please provide a citation that SFV infection is predominately associated with myalgia and arthralgia. SFV is kind of an exception to the rule here.

*We agree and have exchanged MAYV for SFV at that spot in the text.*

5. Line 115: Change alpahviruses to alphavirus

*Fixed as requested*

6. Line 119: Please provide a rationale for making a tree that includes only E1, 6K, and E2 (probably should be written as E2, 6K, and E1). Why not include E3?

*Some alphaviruses like CHIKV, but not all, do not package E3 in the virions so we felt that we did not need to include E3 in the analysis because we were focused on neutralization determinants and accessible antigenic epitopes.*

7. Lime 132: “Interestingly, this person still had heterotypic immunity even at >20 years post infection.” Or was infected with both viruses? Seems like unnecessary speculation.

*We have added the phrase “or as an alternative this person may have undergone infection with the same or a related virus” to indicate that the primary infection immunity could be influenced if the individual was infected again or with a related virus.*

8. Line 150: What is the basis for 56% being considered highly conserved? Compared to VEEV, I suppose it’s true, but compared to other viral proteins, I would guess it’s less conserved

*This would be relative to other more distantly related alphaviruses such as VEEV as the reviewer points out.*

9. Line 161-162: How do you define “long-lasting”?

*We added the phrase “greater than 20 years” to indicate the actual timing.*

10. Please define the orange and blue dots in Fig. 6 in the figure itself.

*The information defining the different colored dots is listed in the title of the Figure 6 legend.*

11. Line 179: I don’t quite understand how both of these statements can be true: “We found that CHIKV and MAYV-MBC frequencies were highly correlated (R2=0.747) **with a ratio of CHIVK:MAYV of about 10:1 overall**” and **“we observed about a four-fold difference in CHIKV versus MAYV MBC frequencies** (9.7 compared to 2.5)”. I imagine I’m understanding something incorrectly. Can you please clarify how these comparisons are different

*We have clarified this in the text as the data shows a four-fold difference.*

12. Line 210: You refer to homotypic immunity. I believe you are referring to homotypic immunity against CHIKV, but please clarify this.

*Added CHIKV as suggested.*

13. “Our data expand on previously published data by Zhang et. al. that identified that amino acids in the E2 A and B domains are involved in the binding of CHIKV to both mouse and human Mxra8, implicating these domains as major targets for therapeutic strategies” I don’t understand the connection here. The current manuscript has nothing to do with Mxra8 other than being a receptor for CHIKV. I don’t understand the inclusion of this sentence.

*We agree and have removed this sentence as it did not contribute to the focus of our manuscript.*

14. The rationale for retaining subject 17 is still unclear. Including this subject in all studies assessing if CHIKV sera generates cross-neutralizing antibodies and MBCs skews the data.

*Prior to our analysis subject 17 was thought to be a CHIKV infection case, inclusion into our study helps to highlight that importance of doing a panel of alphaviruses when trying to identify the infecting virus. Thus, we feel it important to leave this subject in the manuscript. Importantly, the wholistic analysis (Figures 5 and 6D) did not include this subject as we could not conclude that they were a CHIKV subject. Therefore, we added to the methods section “Data from Subject 17 was not included in the analysis represented in Figures 5 and 6D because it is unclear which alphavirus the patient was infected with based upon serology”.*

15. The arrangement of figures and how they are discussed non-chronologically, particularly figure 4 and 5, make the manuscript hard to follow.

*There were probably a couple of approaches to take when describing our results, but we feel that we have chosen a logical progression and are open to specific suggestions.*

16. Figure 2: The cartography is an interesting analysis approach; however, it is a little confusing to follow. I would recommend the author moves this to supplemental figures.

*We respectfully suggest that the antigenic cartography provides a level of visualization that incorporates all of the Neutralization data into a figure and thus, we feel that this is important to display in the manuscript as a figure and not move it to supplemental information.*

17. Figure 3: I recommend that authors remove panels C and D, as the protein structures are not novel information, nor are they easy to understand.

*We feel that showing the protein structures help the reader to visualization the positioning of the E2 B domain relative to the rest of the E2 protein.*

18. Figure 4: I recommend the authors swap the supplemental table that highlights the PRNT50s from these data and the graphs, moving the graphs to supplemental data and the tables to the main text.

*We feel that the graphs help to visualize the data better and we would love to see them stay the way that they are currently.*

19. Figure 5: Panels A-D should be reanalyzed. It is not possible to get a negative number for fold change. I suggest swapping the order of Panels E/F with A-D – this will improve the overall flow.

*As described above there was an error in the excel spreadsheet that has now been fixed and the figures and tables have been updated.*
